# Maternal proximity to mountain-top removal mining and birth defects in Appalachian Kentucky, 1997–2003

**DOI:** 10.1371/journal.pone.0272998

**Published:** 2022-08-11

**Authors:** Daniel B. Cooper, Courtney J. Walker, W. Jay Christian

**Affiliations:** Department of Epidemiology, College of Public Health, University of Kentucky, Lexington, KY, United States of America; Tulane University School of Public Health and Tropical Medicine, UNITED STATES

## Abstract

Extraction of coal through mountaintop removal mining (MTR) alters many dimensions of the landscape. Explosive blasts, exposed rock, and coal washing have the potential to pollute air and water. Previous research suggests that infants born to mothers living in areas with MTR have a higher prevalence of birth defects. In this cross-sectional study, we further examine the relationship between MTR activity and several types of birth defects. Maternal exposure to MTR was assessed using remote sensing data from Skytruth, which captures MTR activity in the Central Appalachian region of the United States. Active MTR area was quantified within a five-kilometer buffer surrounding geocoded maternal address captured on birth records for live births to Appalachian Kentucky mothers between 1997 and 2003 (N = 95,581). We assigned high, medium, and low exposure based on the tertile of total MTR area within 5-km, and births with no MTR within this buffer were assigned zero exposure. The presence or absence of a birth defect grouped into six major organ systems was identified using birth records alone. Finally, we applied conditional multiple imputation for variables with missing values before conducting separate multivariable log-binomial regression models for each birth defect group. Prevalence ratio (PR) estimates were adjusted for individual level covariates from birth records. The prevalence of gastro-intestinal defects was significantly higher in birth records with high and low active MTR exposure compared to records with no exposure. (High exposure: PR = 1.99, 95% CI = 1.14–3.47; low exposure PR = 1.88, 95% CI = 1.06–3.31). This study supports some of the existing findings of previous ecological studies. Research addressing the relationship between gastro-intestinal birth defects and MTR coal mining is warranted but should carefully consider temporal dimensions of exposure.

## Introduction

Mountaintop removal (MTR) is one of many types of surface coal mining used in the Central Appalachian coalfields of the eastern United States, a largely rural region historically dependent on such industries [1]. From 1985 to 2005, the region saw a 250% increase in landscape change from MTR specifically, from 77,000 acres (312 km^2^) in 1985 to 272,000 acres (1101 km^2^) in 2005 [2]. By 2005, approximately 2,900 km^2^ (about 3.5%) of Central Appalachia had been an active surface coal mine at some point [3].

Mountaintop removal coal mining alters the landscape by reducing its topographical complexity, affecting water quality, stream and river flooding, and other physical dimensions of the landscape [4]. To expose buried seams of coal, all or part of a mountain top is removed using heavy machinery and blasting, and excess rock is disposed of in nearby valleys [5]. The exposed rock reacts with rainwater to cause oxidation of metal sulfide minerals, releasing iron, aluminum, cadmium, and copper into surrounding water systems [6]. Slurry ponds, resulting from coal washing, further pollute water sources by releasing arsenic, barium, lead, and manganese [6].

Previous research has shown that the extraction of mineral resources, like coal, is associated with negative health outcomes in humans [7–10]. Congenital birth anomalies, or birth defects, have also been associated with prenatal exposure to environmental contamination [11]. The odds of a birth with congenital heart defects were associated with exposure to oil and gas well activities during early pregnancy in a study of Colorado counties [12], and an increase in the prevalence of neural tube defects was observed among children born to a mother living in areas with natural gas extraction activity [13]. Small et al. found evidence that surface mining was associated with low birth weight in the 2012–2017 period, but not prior to 2000 [14]. This may indicate that there is a lag between debris removal and water contamination, leading to the delayed onset of negative birth outcomes [14]. Additionally, a recently published study found that the intensity of active surface mining near maternal residence during pregnancy was associated with preterm birth and low birth weight [15].

Laboratory and environmental studies of MTR suggest that exposure can occur through contaminated water and air [14, 16]. In a study evaluating air particulate matter at varying distances from surface mining (both traditional and MTR), Kurth et al. reported higher proportions of polycyclic aromatic hydrocarbons (PAHs), silica, and crustal elements like Gallium, Aluminum, Germanium, Rubidium, Lanthanum, and Cerium, in some cases by a factor of 10 or more, compared to control sites that were not located near either surface or underground mining areas [17]. Given the stark contrast between mining and non-mining sites, it is likely that anthropogenic sources, like MTR, were responsible [17].

A previous study of fugitive dust and fumes from MTR mining found that levels of contaminants generated by blasting can be sampled up to 2000 ft (610 meters) away from the blast site, but coal transportation and water runoff may also be a factor [18]. Still, PM collected within one mile (1.6 km) of active MTR has demonstrated potential for adverse health outcomes. In one study, MTR PM promoted cancer growth in human lung cells [19], and in another, cardiac and mitochondrial dysfunction in rats [20]. Birth defects may be associated with MTR coal mining since some substances produced by MTR coal extraction and processing, such as lead, mercury, manganese, iron, selenium, arsenic, chromium, cadmium, ammonium-nitrate, thallium, and PAHs, are associated with an increased risk of developmental and birth anomalies [21].

Ahern et al. hypothesized that a higher prevalence of birth defects would be present in MTR mining counties compared to other coal mining counties and non-mining counties [22]. Their retrospective, ecological study used live births and maternal residence data from four Central Appalachian states, including Kentucky, Tennessee, Virginia, and West Virginia. Using Department of Energy 1996–2003 data, they created a surface mining area map of Central Appalachia, identified MTR sites, and accounted for activity by incorporating county-level coal production figures.

Ahern et al. found that birth defect prevalence rate ratios (PRR) were significantly higher in MTR areas compared to non-mining areas (PRR = 1.26; 95% CI = 1.21–1.32) after controlling for covariates. Six of the seven birth defect groups studied were significantly higher in MTR areas. The PRR remained elevated even after adjusting for socio-economic status (SES), and the spatial analysis suggested that the impacts of mountaintop mining extended beyond the immediate site of mining operations. However, Ahern et al. were limited in their use of a county-level exposure assessment. Notably, counties were classified as MTR coal-mining counties irrespective of the number or size of MTR sites or whether the site was historical or active over the study period.

The present study sought to investigate the relationship between MTR coal mining exposure and birth defects using a finer spatial resolution than in previous literature to assess maternal exposure to MTR. Using a cross-sectional study design, we examined maternal addresses from birth certificates relative to precise satellite data of MTR activity to assess the relationship between congenital anomalies and residential proximity to MTR mining at birth in Appalachian Kentucky.

## Methods

The Medical Institutional Review Board (IRB) at the University of Kentucky and the Kentucky Cabinet for Health and Family Services (CHFS) IRB approved this study protocol (Protocol #50352, Approved April 30, 2019).

### Data sources and study population

The Kentucky Department for Public Health, Office of Vital Statistics, provided individual outcome and covariate data from birth records, which included all live births to Kentucky residents from January 1, 1997, to December 31, 2003, using birth certificate formatting of the 1989 revision to U.S. standard live birth certificate. The study period of 1997 to 2003 was chosen because it precedes Kentucky’s adoption of a new standard in 2004, to increase comparability of births within the dataset, and to increase comparability with previous studies. Requested records identified maternal residential address, congenital anomalies, and covariate data.

Among all live births between 1997 and 2003 (N = 376,092), 24,849 (6.6%) self-reported maternal addresses could not be geocoded, and 255,444 (67.9%) were not Appalachian (defined by the Appalachian Regional Commission [23]). Further, implausible birth weights (<500 grams or >5500 grams; n = 189) [24], implausible gestational ages (<22 weeks or >44 weeks; n = 28) [24, 25], and five births with unknown infant sex at birth were excluded, resulting in 95,581 live births from January 1, 1997, to December 31, 2003, for this study.

Mountain-top removal coal mining polygons were sourced from a dataset produced by SkyTruth.org, a non-profit that tracks resource extraction using remote sensing data. This data set includes several GIS layers representing active MTR mining sites in the Appalachian region by year. It possesses both high temporal and spatial scale and is made available freely and openly by its original creators to facilitate future research [3]. Their technique identified areas that were both included in mining permits and showed substantial changes in topography compared to the previous year, as observed in remote sensing (i.e., satellite) imagery data. Pericak et al. have further described methods for producing this dataset [3].

### Exposure variables

ArcGIS (v10.4.1; Redlands, CA) software was used to determine exposure to active MTR. MTR mining polygons with an area <40 acres (0.12 km^2^) were first excluded from further processes due to the improbability of being a viable surface mining area [26]. We assigned each birth record a year of exposure based on when the majority of pregnancy occurred (>50%, including a 12-week pre-conception period). This pre-conception period of 12 weeks prior to the last menstrual period (LMP) was estimated using the gestational age at birth, as gestational age is based off the LMP. The year of exposure assigned determined the year of active MTR to use in assessing exposure, and the residence they requested the birth certificate be sent was the only geocoded location used to measure exposure to surrounding active MTR sites.

**Fig 1:**
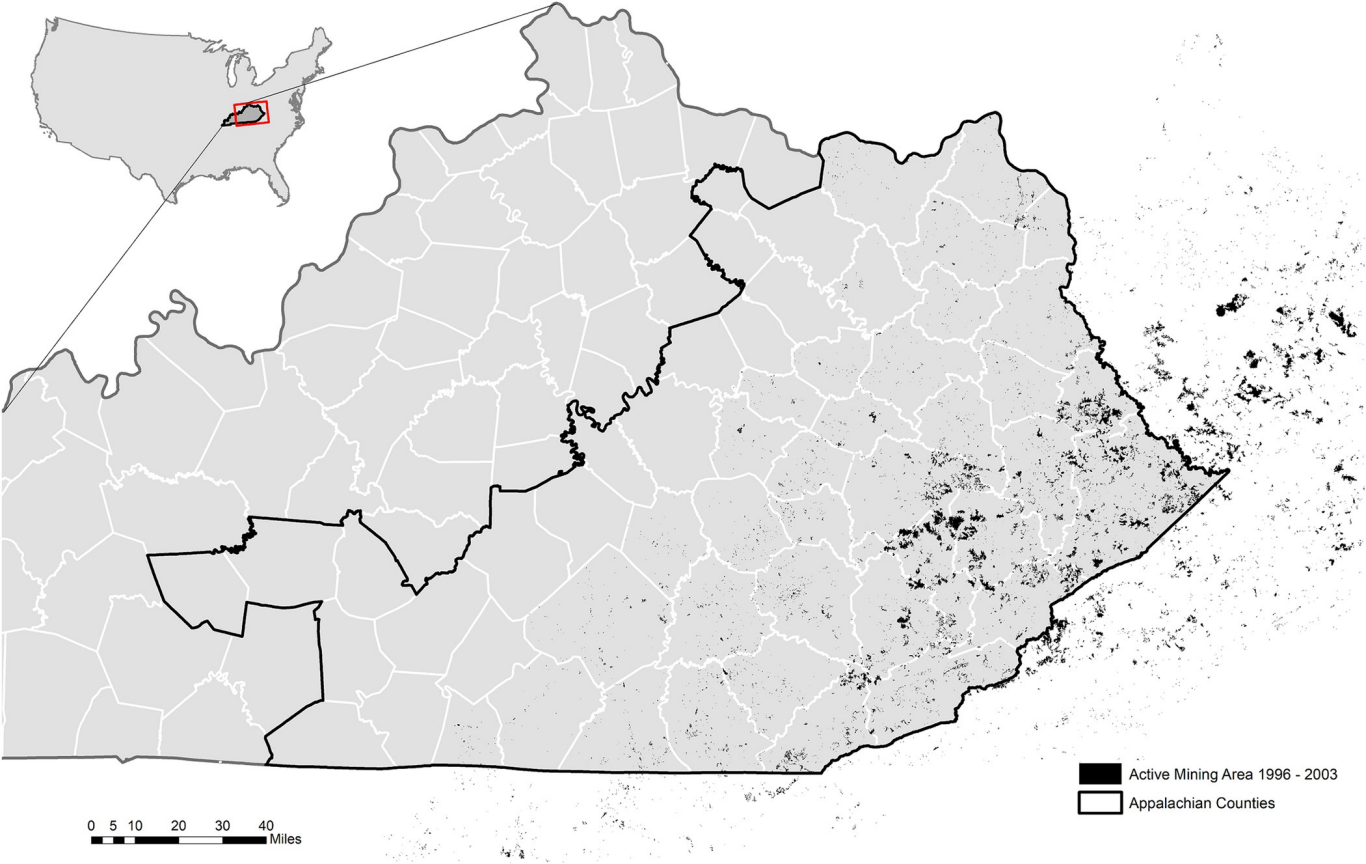
Cumulative active mountain top removal mining areas from 1996–2003 overlaying Kentucky Appalachian designated counties.

Addresses were matched to either a precise point location or to the street, city, or ZIP (United States Postal Service ’Zone Improvement Plan’ area) code centroid, depending on the strength of the match. Geocoding match scores (or level of agreement) less than 93% were unsuccessfully matched, and 93.3% of all addresses geocoded to the ZIP code or better. The spatial join tool computed the total area of active MTR within a 5-kilometer (km) buffer surrounding the matched location. At least one previous study of birth outcomes uses a similar measure of exposure [15]. This process was repeated for all exposure years in the study population, each time matching the appropriate year of active MTR mining to each record. Fig 1 identifies the study region of Appalachian Kentucky counties and provides a cumulative map of MTR mining during the study period.

The final exposure variable was categorized into low, medium, and high MTR exposure based on tertiles of active MTR area within 5-km of maternal residence. Births where maternal residence was not within 5-km of active MTR were given a 0 (no exposure). Births in the low tertile of exposure had up to 0.32 square km of active MTR within the 5-km buffer, medium >0.32 to 1.17 square km, and high >1.17 square km. We categorized the exposure variable after examining the non-normal distribution of active MTR area near maternal residence and the considerable proportion of births in the population with zero exposure.

### Covariates and outcome variables

Birth records included twenty-two congenital anomalies grouped into six major organ systems. The central nervous system group included anencephaly, spina bifida, hydrocephaly, and macrocephaly; circulatory/respiratory group: heart malformations; gastro-intestinal group: omphalocele/ gastroschisis and tracheoesophageal fistula; urogenital group: genital malformation and renal agenesis; musculoskeletal group cleft lip, clubfoot, diaphragmatic hernia, and polydactyly, syndactyly, and adactyly; and chromosomal group: Down syndrome. All groups also include nonspecific categories. This approach was also taken by Ahern et al [22]. For each group, the variable was categorized as (1) presence of a birth defect within that group or (0) absence of any birth defect. An additional dichotomous variable—presence or absence of any congenital anomaly—was also created. Birth defect groups were not limited to isolated cases of the defect, as this would greatly reduce our counts within each group. This results in some births with defects in multiple groups.

Individual-level covariates originated from birth record demographic variables. Categorical definitions were based on meaningful qualitative cut points identified through literature. Infant covariates included male or female sex, gestational age at delivery (<37, 37–39, ≥40 weeks), and birth weight (<1500, 1500–2499, 2500–3999, ≥4000 grams). Maternal covariates included education (0–11 years education, 12 years, and ≥13 years), age at delivery (< 20, 20–34, ≥35), any diabetes comorbidity (including Type II), cigarette use, parity (previous births), plurality (singleton, twin or more), and Kotelchuck index of prenatal care (inadequate, intermediate, adequate, adequate+) [27].

### Statistical analyses

All birth defect groups were dependent variables in separate multivariable log-binomial regression models used to estimate prevalence ratios (PRs) and 95% confidence intervals (CIs). Covariates included in the final statistical models were based on previous literature. All final models included maternal education [28], age of mother [28, 29] cigarette smoking [28], diabetes comorbidity [30], Kotelchuck index of prenatal care adequacy, and sex of infant [31]. Chi-square tests for association and regression models were implemented in R (v4.1.1) [32] using RStudio (v2021.9.0.351) [33], and p-values less than 0.05 were considered statistically significant. In adjusted models, we applied conditional multiple imputation for variables with missing values using the mice R package (v3.14.0) [34]. Adjusted estimates were pooled from five conditionally imputed datasets, which reduces potential bias from using complete case analysis and increases the number of births included in analyses with defects in categories where small numbers are present.

## Results

Table 1 examines birth characteristics by exposure to active MTR mining. Among all live births, 35.6% (34,006/ 95,581) were exposed to MTR within 5-km of maternal residence. MTR exposed mothers were slightly younger than unexposed (none: 16.8% < age 20; low exposure: 17.4%; medium: 18.5%; high 18.2%), more often non-Hispanic white (none: 97.2%; low: 98.5%; medium: 98.6%; high: 99%), slightly less educated (none: 27.2% less than high school; low: 27.9%; medium: 31%; high: 29.9%), and were more often tobacco users (none: 28.9%; low: 30.8%; medium: 33.2%; high: 30.9%). There were also significant differences in adequacy of prenatal care. Unexposed mothers more often had adequate care (49.8%) compared to mothers with low exposure (47.1%), medium (43.7%), and high (44.7%). A higher proportion of exposed births had lower gestational age at delivery compared to unexposed (none: 40.5% >39 weeks; low: 36.9%; medium: 37.8%; high: 37.1%). Finally, exposed birth records more often had geocoded maternal address match to the ZIP code level (low: 42.6%; medium: 54.7%%; high: 50.8%%) compared to records with no exposure (29.6%). See Table A in S1 Text for an examination of the distribution of maternal and birth characteristics by birth defect groups.

**Table 1:** Characteristics of Appalachian Kentucky birth records from 1997 to 2003 by level of exposure to active mountain-top removal mining within five kilometers of maternal residence reported at birth (N = 95,581).

Characteristic, n (%)	None (n = 61575)	Low (n = 11336)	Medium (n = 11335)	High (n = 11335)	p-value^a^
Sex of child					
Male	31,591 (51.3)	5,836 (51.5)	5,929 (52.3)	5,862 (51.7)	0.25
Female	29,984 (48.7)	5,500 (48.5)	5,406 (47.7)	5,473 (48.3)	
Gestational age at delivery (weeks)					
<37	6,499 (10.6)	1,233 (10.9)	1,178 (10.4)	1,210 (10.7)	<0.001
37–39	30,124 (48.9)	5,924 (52.3)	5,875 (51.8)	5,925 (52.3)	
>39	24,952 (40.5)	4,179 (36.9)	4,282 (37.8)	4,200 (37.1)	
Birth weight at delivery (grams)					
<1500	804 (1.3)	141 (1.2)	152 (1.3)	137 (1.2)	0.60
1500–2500	4,165 (6.8)	798 (7.0)	774 (6.8)	816 (7.2)	
>2500	56,606 (91.9)	10,397 (91.7)	10,409 (91.8)	10,382 (91.6)	
Age of mother at delivery					
<20	10,348 (16.8)	1,968 (17.4)	2,096 (18.5)	2,057 (18.2)	<0.001
20–34	47,134 (76.6)	8,716 (76.9)	8,583 (75.7)	8,647 (76.3)	
>34	4,061 (6.6)	645 (5.7)	652 (5.8)	627 (5.5)	
Missing	32	7	4	4	
Race of mother					
White non-Hispanic	59,793 (97.2)	11,147 (98.5)	11,164 (98.6)	11,212 (99.0)	<0.001
Other	1,703 (2.8)	174 (1.5)	158 (1.4)	113 (1.0)	
Missing	79	15	13	10	
Education of mother at delivery					
Less than high school	16,754 (27.2)	3,161 (27.9)	3,510 (31.0)	3,385 (29.9)	<0.001
High school	24,692 (40.1)	4,539 (40.1)	4,479 (39.6)	4,360 (38.5)	
More than high school	20,063 (32.6)	3,622 (32.0)	3,330 (29.4)	3,570 (31.6)	
Missing	66	14	16	20	
Maternal tobacco use	17,219 (28.9)	3,403 (30.8)	3,667 (33.2)	3,379 (30.9)	<0.001
Missing	1,974	300	299	416	
Diabetes in mother	2,013 (3.3)	363 (3.2)	351 (3.1)	360 (3.2)	0.78
Previous births					
No previous births	26,481 (43.2)	4,887 (43.3)	4,875 (43.2)	5,045 (44.7)	0.027
At least one previous birth	34,844 (56.8)	6,402 (56.7)	6,412 (56.8)	6,241 (55.3)	
Missing	250	47	48	49	
Plurality					
Singleton	59,856 (97.2)	11,037 (97.4)	11,060 (97.6)	11,032 (97.4)	0.13
Twin or more	1,705 (2.8)	294 (2.6)	273 (2.4)	294 (2.6)	
Missing	14	5	2	9	
Kotelchuck Index					
Inadequate	6,265 (10.5)	1,155 (10.5)	1,226 (11.1)	1,186 (10.7)	<0.001
Intermediate	14,869 (24.8)	2,944 (26.7)	3,157 (28.7)	2,897 (26.2)	
Adequate	29,842 (49.8)	5,183 (47.1)	4,819 (43.7)	4,956 (44.7)	
More than Adequate	8,892 (14.9)	1,725 (15.7)	1,815 (16.5)	2,037 (18.4)	
Missing	1,707	329	318	259	
Address match level					
Address point	22,425 (36.4)	2,932 (25.9)	2,146 (18.9)	1,492 (13.2)	<0.001
Street address range	20,591 (33.4)	3,526 (31.1)	2,923 (25.8)	3,987 (35.2)	
Zip code centroid	18,233 (29.6)	4,828 (42.6)	6,202 (54.7)	5,756 (50.8)	
City centroid	326 (0.5)	50 (0.4)	64 (0.6)	100 (0.9)	

Low exposure: Up to 0.32 km^2^; medium: >0.32–1.17 km^2^; high > 1.17 km^2^

ZIP = United States Postal Service ’Zone Improvement Plan’ area

^a^ Chi-squared test comparing characteristic frequencies by level of exposure

Table 2 summarizes the relationship between exposure to active MTR within 5-km of maternal residence and birth defect outcomes. Among all live births, 98.6% had no birth defect reported on the birth record and 1.37% had at least one birth defect. See Table B in S1 Text for a brief examination of births with isolated cases versus those with defects in multiple organ systems. Significantly fewer births with central nervous (59.6%) or gastro-intestinal (50.0%) defects had zero active MTR exposure compared to births with no defect (64.4%). Additionally, a larger proportion of births with central nervous (16.9%) or gastro-intestinal (18.5%) defects had high exposure to active MTR compared to births with no defect (11.9%). There were no other statistically significant differences in active MTR exposure between births with defects in other system groups compared to births with no defects.

**Table 2:** Birth defect outcomes by level of exposure to active mountain-top removal mining within five kilometers of maternal residence.

Group, n (%)	Overall (N = 95,581)	None (n = 61,575)	Low (n = 11,336)	Medium (n = 11,335)	High (n = 11,335)	p-value^a^
Any defect^b^	1,311	828 (63.2)	160 (12.2)	161 (12.3)	162 (12.4)	0.82
Central nervous	89	53 (59.6)	5 (5.6)	16 (18.0)	15 (16.9)	0.047
Circulatory/ respiratory	266	171 (64.3)	36 (13.5)	31 (11.7)	28 (10.5)	0.79
Gastro-intestinal	92	46 (50.0)	16 (17.4)	13 (14.1)	17 (18.5)	0.027
Urogenital	288	179 (62.2)	36 (12.5)	41 (14.2)	32 (11.1)	0.61
Musculo-skeletal	577	374 (64.8)	67 (11.6)	67 (11.6)	69 (12.0)	>0.99
Chromosomal	95	64 (67.4)	13 (13.7)	9 (9.5)	9 (9.5)	0.73
No defect	94,270	60,747 (64.4)	11,176 (11.9)	11,174 (11.9)	11,173 (11.9)	--

Low exposure: Up to 0.32 km^2^; medium: >0.32–1.17 km^2^; high > 1.17 km^2^

^a^ Chi-squared test comparing frequency of defect by level of exposure

^b^ Birth defect groups will not add up to match the any defect group as some births were reported to have multiple defects

Table 3 displays crude and adjusted PRs for each birth defect outcome. Most birth record covariates were retained as they are known risk factors for birth defects. Although pregestational diabetes could not be determined separately from gestational diabetes, the covariate was retained in the model as pregestational diabetes is a known risk factor for some birth defects and the term was significant after adjustment in multiple models. Birth weight and gestational age at birth were not included during modelling as low birth weight and preterm birth share similar risk factors with congenital anomalies, and congenital anomalies may be a risk factors for low birth weight and preterm birth [35]. Address level match was not significantly associated with birth defect groups, so it was not retained in the final model. Finally, parity was not retained as the number of previous births is interrelated with maternal age, adding noise to the model.

**Table 3:** Separate log-binomial models estimating the crude and adjusted relationship between active mountain top removal mining exposure within 5-km of maternal address and presence of a birth defect.

	Crude		Adjusted^a^	
Model Outcome/ Exposure Level	PR	95% CI	PR	95% CI
Any defect				
No MTR within 5km	1.00	Reference	1.00	Reference
Low exposure	1.05	0.88–1.24	1.05	0.88–1.24
Medium exposure	1.06	0.89–1.25	1.04	0.88–1.23
High exposure	1.06	0.90–1.25	1.06	0.89–1.25
Central nervous				
No MTR within 5km	1.00	Reference	1.00	Reference
Low exposure	0.51	0.18–1.16	0.52	0.21–1.29
Medium exposure	1.64	0.91–2.80	1.66	0.95–2.90
High exposure	1.54	0.84–2.66	1.56	0.88–2.76
Circulatory/ respiratory				
No MTR within 5km	1.00	Reference	1.00	Reference
Low exposure	1.14	0.79–1.62	1.14	0.79–1.63
Medium exposure	0.99	0.66–1.42	0.97	0.66–1.42
High exposure	0.89	0.59–1.30	0.88	0.59–1.31
Gastro-intestinal				
No MTR within 5km	1.00	Reference	1.00	Reference
Low exposure	1.89	1.04–3.26	1.88	1.06–3.31
Medium exposure	1.54	0.80–2.76	1.49	0.81–2.77
High exposure	2.01	1.12–3.43	1.99	1.14–3.47
Urogenital				
No MTR within 5km	1.00	Reference	1.00	Reference
Low exposure	1.09	0.75–1.54	1.09	0.76–1.55
Medium exposure	1.24	0.88–1.73	1.21	0.86–1.70
High exposure	0.97	0.66–1.39	0.96	0.66–1.40
Musculo-skeletal				
No MTR within 5km	1.00	Reference	1.00	Reference
Low exposure	0.97	0.75–1.25	0.97	0.75–1.26
Medium exposure	0.97	0.75–1.25	0.96	0.74–1.25
High exposure	1.00	0.77–1.29	1.00	0.77–1.29
Chromosomal				
No MTR within 5km	1.00	Reference	1.00	Reference
Low exposure	1.10	0.58–1.94	1.11	0.61–2.02
Medium exposure	0.76	0.35–1.46	0.76	0.38–1.53
High exposure	0.76	0.35–1.46	0.77	0.38–1.55

PR = Prevalence Ratio; CI = Confidence Interval

^a^Adjusted for maternal education, age, tobacco use, diabetes comorbidity, Kotelchuck index, plurality, and sex of the infant. Adjusted PR estimates are pooled from five conditionally imputed datasets.

We found only gastro-intestinal defects to be more prevalent among births with MTR exposure during unadjusted analysis. After adjusting for maternal education, age, tobacco use, diabetes comorbidity, Kotelchuck index, plurality, and sex of the infant in the log-binomial regression model, the relationship between MTR exposure and gastro-intestinal defects remained statistically significant. The prevalence of gastro-intestinal defects was 88% higher in birth records with low exposure to active MTR within 5-km of residence compared to records with no exposure (PR = 1.88; 95% CI = 1.06, 3.31). The prevalence of gastro-intestinal defects was 99% higher in birth records with high exposure compared to records with no exposure (PR = 1.99; 95% CI = 1.14, 3.47).

Since mothers 25 years of age and younger are more likely to have a baby with gastroschisis compared to older mothers, we examined whether age modified the relationship between MTR exposure and prevalence of gastro-intestinal defects [36, 37]. In stratified analysis, the increase in gastro-intestinal defect prevalence did not remain significant in mothers 25 years of age or younger, although the estimate still trended in the same direction. The PR for mothers 26 or older with MTR exposure increased compared to the crude analysis (See Table C in S1 Text). In addition, the statistical interaction between age and MTR exposure was insignificant in both the full crude and adjusted gastro-intestinal model, so the interaction term was not retained. Maternal age did not modify the relationship between MTR exposure and prevalence of gastro-intestinal defects.

## Discussion

We observed that gastro-intestinal birth defects were associated with maternal residential proximity to MTR mining. Before or after controlling for individual-level covariates, no other birth defect group was associated with MTR activity. We did not find that differences in covariates between the exposed and unexposed populations contributed to significant birth defect prevalence differences, as estimates changed little when literature-based covariates were incorporated into the model. Our results show an increase in the prevalence of gastro-intestinal defects in births with geocoded maternal residence within 5-km of active MTR in Appalachian Kentucky counties. After quantifying the amount of active MTR area within the 5-km buffer, birth records with the lowest and highest exposure had increased prevalence of gastro-intestinal defects compared to records with no exposure. While not significant, the prevalence in the medium exposure group still trended in the same direction. A preliminary analysis using a binary indicator of MTR exposure (home address within 1 km of active MTR sites) showed a similar increase in the prevalence of gastro-intestinal defects.

Although we only examine a subset of Central Appalachian counties located in the state of Kentucky, the United States’ Appalachian region consists of 13 states and 423 counties that span from New York to Georgia [38]. The homogeneity of this region may reduce our ability to generalize to other groups, but we believe that our more defined population, which limits not only hospital differences but other potential environmental exposures, allows us an opportunity to better explore active MTR exposure associations with poor birth outcomes.

Two previous studies examined the relationship between MTR surface mining and adverse birth outcomes in this region [22, 39]. Ahern et al. found significantly higher prevalence of all birth defects groups, except for chromosomal, in MTR mining areas compared to non-mining areas. We only found support for an increase in the prevalence of gastro-intestinal defects. Lamm et al. found that adjustment for the hospital of birth explained the relationship between MTR mining exposure and birth defects, possibly due to variability in birth certificate reporting practices introducing differential misclassification of defects on the birth record. Our study reduced some variability in birth certificate reporting by restricting birth records to a relatively homogeneous region of a single state (i.e., Appalachian counties in Kentucky); however, neither the present analysis nor a similar analysis by Buttling et al. adjusted for the hospital of birth, so the confounder cannot be ruled out [40, 41].

Still, our more precise exposure assessment should reduce possible differential misclassification bias. While Lamm et al. rejected the Ahern et al. hypothesis, both studies shared a county-level exposure assessment, which might obscure the true relationship between MTR mining and birth defects. In our study, individuals from the same county could have different assessed exposures but still share a birth hospital. Our approach was like that of Buttling et al., where satellite images of surface mining and maternal street address and ZIP code were used to quantify mining area within a 5-km buffer of maternal residence [15]. The use of this methodology resulted in a finer spatial resolution assessment of maternal exposure to mining sites. Buttling et al. found that the amount of active surface mining (MTR and other forms of surface mining) area near maternal residence during pregnancy was associated with other adverse birth outcomes, such as preterm birth and low birth weight, both of which have similar risk factors to congenital anomalies [35].

Our study also improves on existing literature by recognizing the potential for exposure during the pre-conception period (12 weeks prior to estimated LMP), as some trace elements and heavy metals (Cd, As, Cr, Hg, & Pb) bioaccumulate, can cross the placental barrier, and are associated with stillbirth, impairments in the development of the central nervous system, and maternal complications [42]. Our study had no environmental or biological data to investigate actual MTR contaminant exposure, but previous research has shown dust from MTR sites contains high concentrations of crustal elements, PAHs, and silica [17]. Laboratory studies using MTR PM from similar distances used in our study demonstrated biological adverse events in human lung cells and in rats [19]. Various environmental contaminants from coal mining have previously been suggested as agents associated with reproductive issues like birth defects [43, 44]. Further, paternal occupational mining exposure may increase the likelihood of births defects, although the mechanism of this association is unclear [45]. As birth records do not capture parental occupation, we were unable to adjust for this potential source of exposure.

However, previous literature is inconclusive regarding the distance MTR-derived agents might travel. Exposure pathways, duration, and quantity may be heterogeneous within the study population due to variation in mining practices, geography, coal transportation, chemical treatment, and weather, as seen in Kurth et al. [17]. Our quantification of active MTR mining surrounding maternal residence helps to address this, since the measure considers the number of active MTR sites and their size. However, our study only examined active MTR mining and could not assess exposure to historic/ inactive sites. It is important that future research further investigates these mechanisms of exposure.

As our study relied on birth records alone to identify maternal information and birth outcomes, self-reported tobacco use and residential address may be inaccurate due to recall or interviewer bias. Similarly, spelling, address truncation, and other address errors can impact the accuracy of geocoding and thus exposure assessment. Spatially non-random differences in the success of geocoding have been demonstrated previously [46], and geocoding results are more accurate in urban than rural areas [47]. Our geocoding results when matching maternal addresses demonstrated the difficulty in identifying precise address point location used during exposure assessment. A sizable proportion (30.1%) of our birth records were matched to a ZIP code or city centroid, introducing possible exposure misclassification for those records. Buttling et al. chose to conduct separate analyses at the ZIP code level for all records and at the street address level for records with a more precise address match. We conducted a single analysis, where 69.8% of records matched to a more precise location (i.e., street or point level). While geographic bias may be increased due to rurality, misclassification of exposure is likely non-differential for this study, as all addresses come from Appalachian counties in Kentucky and address match level was not associated with birth defect outcomes.

Differences in maternal characteristics may impact rates of termination, such as access to adequate prenatal care or socio-economic status, age, race/ ethnicity, urban vs rural residence, and previous pregnancy [48, 49]. We identified a significant difference in the proportion of records among the levels of MTR exposure with regards to prenatal care adequacy, so we cannot rule out the possibility of a bias away from the null if exposed pregnancies were less likely to be electively terminated due to these maternal factors. Additionally, we could not assess successive births from the same mother due to the deidentification of the data. This would have allowed us to examine birth order as an additional covariate and to cluster our models by mother to increase their accuracy, since births from the same mother are not truly independent observations during analysis. However, given that our study population was limited to Appalachian counties in Kentucky, differences in covariates between exposed and unexposed are minimal, as demonstrated by the insignificant change in PR estimates after the inclusion of individual characteristics in statistical models.

Bell and Belanger identified the residential mobility of the mother as a key determinant of environmental exposures during pregnancy [50]. However, in an assessment of Texas Birth Defects registry data (1997–2000), Canfield et al. reported non-differential bias. Although no such information was available for this study, the Appalachian region has historically had low residential mobility, although many studies focus on a larger scale of mobility, rather than movement within a community [51]. We are limited in our ability to interpret a causal relationship between MTR activity and birth defects or examine this potential for exposure misclassification since longitudinal data like this were unavailable.

As we could only access live birth records, we did not capture all congenital anomalies within the population. Watkins et al. demonstrated that the sensitivity of birth certificates in reporting birth defects is overall low and varies by type due to ease of detection at birth [52], and prenatal diagnosis of birth defects can lead to termination of pregnancy, affecting the results of epidemiological research [48]. We also could not examine stillbirths in this population, as stillbirths were not available electronically in Kentucky until 2000. We nevertheless used this source to identify birth defects to compare our results more directly with those of Ahern et al. and Lamm et al. Furthermore, we eliminated records with implausible birthweights and gestational ages, which could have introduced some bias. However, of those excluded due to implausibility, we identified only one record with a congenital structural defect. This record was unexposed to MTR, reducing our concerns regarding bias in this context.

We observed a slightly lower but similar prevalence of birth defects among live births compared to Ahern et al. and Lamm et al [22, 39]. The prevalence of any birth defect in the Ahern et al. study was 1.52% in Central Appalachian states (All counties in Kentucky, Tennessee, Virginia, and West Virginia) from 1996–2003, compared to 1.37% in our study of only Appalachian-designated counties in Kentucky from 1997–2003. Lamm et al. reported a 1.82% prevalence from 1990 through 2009 in West Virginian counties alone. While Buttling et al. did not examine birth defects, they examined related birth outcomes in Appalachian counties in Central Appalachia, including Kentucky, from 1990–2015, which also overlaps with the present study’s geographic location extent and periods.

Studies using birth defects registries have estimated the prevalence of any birth defect at about 3% in Kentucky and the U.S. [53]. Consistent with prior literature using birth records alone, we found fewer records with congenital birth defects than likely prevalent in the study population. This limits statistical power and could result in bias due to misclassification of outcome. The association we observed between maternal MTR exposure and gastro-intestinal defects, for example, was based on 46 exposed records with congenital defects. However, since identification and diagnosis of abdominal wall defects, such as omphalocele and gastroschisis, commonly occurs through prenatal ultrasound, and these gastro-intestinal defects are visible during a physical examination at birth [54], we believe this is less of a concern for this outcome. It is more likely that bias was introduced by the inability to assess terminated pregnancies for these defects. Still, we believe any such bias would likely be non-differential, as there is not sufficient evidence that exposure status would influence whether birth defects were captured on the birth certificate, and rural women, which compose much of the study population, have additional barriers to elective termination [40, 55]. Furthermore, our analysis showed a relationship between maternal smoking and cleft lip/palate, a well-known association that provides some validation [56]. Future research may reduce under-detection or misdiagnosis of birth defects by utilizing alternative data sources, such as birth defects registries, and including pregnancies that ended in stillbirth and electively terminated pregnancies.

Despite these limitations, our study builds upon previous research by quantifying active MTR exposure for all birth records individually and at a finer spatial scale compared to the county-level assessments in previous literature examining the relationship between MTR and birth defects.

## Conclusion

This study found that MTR near maternal residence reported at birth was associated with the presence of gastro-intestinal birth defects in infants born to women in Appalachian Kentucky between 1997 and 2003. Taking into consideration the amount of active MTR surface mining land within a 5-km buffer of maternal residence during the year of majority exposure during pregnancy, we found that the prevalence of gastro-intestinal defects was significantly higher in birth records with high and low active MTR exposure compared to records with no exposure. This finding agrees and contrasts with previous research that demonstrated evidence of an association between several additional birth defects in MTR mining areas. Based on our study’s results, additional research addressing the relationship between gastro-intestinal birth defects and MTR coal mining is warranted. Additional research is needed at this spatial scale of exposure with better adjustments for data heterogeneity, such as hospital of birth, maternal residential history, occupational characteristics of household members, a more robust source of birth defect cases, and the inclusion of electively terminated pregnancies. Furthermore, research is needed to better characterize the type and amount of environmental contamination encountered by communities in close proximity to active MTR sites since prior studies have not conclusively examined this issue.

## Supporting information

S1 Text Supporting tables for birth defects by birth characteristics, examination of births with multiple defects, and age-stratified analysis. (PDF)
