# Supplementary material for: Maternal proximity to mountain-top removal mining and birth defects in Appalachian Kentucky, 1997–2003
Source: PLoS One. 2022 Aug 11;17(8):e0272998. doi: 10.1371/journal.pone.0272998 (PMC9371306; doi:10.1371/journal.pone.0272998)
Supplement: S1 Text — (PDF) [file pone.0272998.s001.pdf]

Supporting text and tables

Table A summarizes participant characteristics in relation to the presence of congenital defects. There were 95,581 birth records included in the analysis from Appalachian-Kentucky counties and 1311 (13.7 per 1000 live births) total births with any congenital anomaly. Musculoskeletal congenital defects were the most prevalent over the study period (n = 577, 6.0 per 1000 live births). Race was the only characteristic with no significant differences between births with a birth defect and births without. Maternal education in births with congenital defects was generally lower than those without defects (28.0% less than high school) with significant differences in the any (30.9% less than high school), urogenital (34.1% less than high school), and musculoskeletal (30.9% less than high school) groups. Births with no defects had a lower proportion of mothers aged 34 or older (6.3 %) compared to circulatory/respiratory (9.4%), urogenital (7.6%) and chromosomal (22.3%) groups. Infant sex was generally the same for those with and without defects, except for the urogenital and any defect groupings, where there were significantly more males (urogenital 82.6%; any defect 58%; no defect 51.4%). Tobacco use during pregnancy was mostly similar for those with and without congenital defects, although a significant difference was detected among musculoskeletal and any defect groups. There was a significant ($p < 0.001$) difference between births with no defect and all groups with respect to gestational age at delivery and birth weight. Births with congenital defects generally had lower gestational age at delivery and weighed less than those with no defects. In general, a larger proportion of records with congenital defects had inadequate prenatal care than records with no defects, except for the chromosomal group. Births with chromosomal defects also had a higher proportion of mothers with a previous birth (68.4%) compared to births with no defects (56.8%), and the musculoskeletal group had a higher proportion of mothers with no previous births (47.y%; no defects 43.4%).

Table A. Birth characteristics of Appalachian Kentucky births from 1997 to 2003 by defect type (N=95,581)

Characteristic, n (%)	No defect (n = 94,270)	Any defect (n = 1,311)[a]	p-value	Central nervous (n = 89)	p-value	Circulatory/ respiratory (n = 266)	p-value	Gastro-intestinal (n = 92)	p-value	Urogenital (n = 288)	p-value	Musculo-skeletal (n = 577)	p-value	Chromosomal (n = 95)	p-value
Sex of child			<0.001		0.71		0.31		0.37		<0.001		0.59		0.070
Male	48,458 (51.4)	760 (58.0)		44 (49.4)		145 (54.5)		43 (46.7)		238 (82.6)		303 (52.5)		40 (42.1)	
Female	45,812 (48.6)	551 (42.0)		45 (50.6)		121 (45.5)		49 (53.3)		50 (17.4)		274 (47.5)		55 (57.9)	
Gestational age at delivery			<0.001		<0.001		<0.001		<0.001		<0.001		<0.001		<0.001
<37	9,871 (10.5)	249 (19.0)		26 (29.2)		51 (19.2)		36 (39.1)		52 (18.1)		92 (15.9)		23 (24.2)	
37-39	47,187 (50.1)	661 (50.4)		51 (57.3)		137 (51.5)		43 (46.7)		133 (46.2)		293 (50.8)		52 (54.7)	
>39	37,212 (39.5)	401 (30.6)		12 (13.5)		78 (29.3)		13 (14.1)		103 (35.8)		192 (33.3)		20 (21.1)	
Birth weight at delivery (grams)			<0.001		<0.001		<0.001		<0.001		<0.001		<0.001		<0.001
<1500	1,198 (1.3)	36 (2.7)		5 (5.6)		6 (2.3)		2 (2.2)		10 (3.5)		16 (2.8)		5 (5.3)	
1500-2500	6,398 (6.8)	155 (11.8)		16 (18.0)		34 (12.8)		26 (28.3)		32 (11.1)		57 (9.9)		13 (13.7)	
>2500	86,674 (91.9)	1,120 (85.4)		68 (76.4)		226 (85.0)		64 (69.6)		246 (85.4)		504 (87.3)		77 (81.1)	
Age of mother at delivery			0.30		0.63		0.052		0.56		0.054		0.47		<0.001
<20	16,240 (17.2)	229 (17.5)		18 (20.2)		37 (13.9)		19 (20.7)		63 (21.9)		98 (17.0)		9 (9.6)	
20-34	72,095 (76.5)	985 (75.2)		67 (75.3)		204 (76.7)		66 (71.7)		203 (70.5)		449 (78.0)		64 (68.1)	
>34	5,890 (6.3)	95 (7.3)		4 (4.5)		25 (9.4)		7 (7.6)		22 (7.6)		29 (5.0)		21 (22.3)	
Missing	45	2		0		0		0		0		1		1	
Race of mother			0.39		>0.99		0.10		>0.99		0.54		0.089		>0.99
White non-hispanic	92,041 (97.8)	1,275 (97.4)		87 (97.8)		264 (99.2)		90 (97.8)		279 (97.2)		557 (96.7)		93 (97.9)	
Other	2,114 (2.2)	34 (2.6)		2 (2.2)		2 (0.8)		2 (2.2)		8 (2.8)		19 (3.3)		2 (2.1)	
Missing	115	2		0		0		0		1		1		0	
Education of mother at delivery			<0.001		0.22		0.37		0.36		0.031		0.026		0.82
Less than high school	26,405 (28.0)	405 (30.9)		27 (30.3)		74 (27.8)		32 (34.8)		98 (34.1)		178 (30.9)		24 (25.3)	
High school	37,523 (39.9)	547 (41.8)		41 (46.1)		116 (43.6)		33 (35.9)		114 (39.7)		243 (42.2)		40 (42.1)	
More than high school	30,228 (32.1)	357 (27.3)		21 (23.6)		76 (28.6)		27 (29.3)		75 (26.1)		155 (26.9)		31 (32.6)	
Missing	114	2		0		0		0		1		1		0	
Maternal tobacco use	27,224 (29.8)	444 (34.5)	<0.001	19 (21.6)	0.092	88 (34.0)	0.14	33 (36.3)	0.18	94 (33.5)	0.18	207 (36.6)	<0.001	30 (32.6)	0.56

Table A. Birth characteristics of Appalachian Kentucky births from 1997 to 2003 by defect type (N=95,581)

Characteristic, n (%)	No defect (n = 94,270)	Any defect (n = 1,311)[a]	p-value	Central nervous (n = 89)	p-value	Circulatory/ respiratory (n = 266)	p-value	Gastro-intestinal (n = 92)	p-value	Urogenital (n = 288)	p-value	Musculo-skeletal (n = 577)	p-value	Chromosomal (n = 95)	p-value
Missing	2,964	25		1		7		1		7		11		3	
Diabetes in mother	3,014 (3.2)	73 (5.6)	<0.001	6 (6.7)	0.066	24 (9.0)	<0.001	1 (1.1)	0.38	10 (3.5)	0.79	35 (6.1)	<0.001	2 (2.1)	0.77
Previous births			0.32		0.73		0.43		0.82		0.33		0.034		0.021
No previous births	40,702 (43.4)	586 (44.7)		37 (41.6)		109 (41.0)		41 (44.6)		133 (46.2)		275 (47.7)		30 (31.6)	
At least 1 previous birth	53,175 (56.6)	724 (55.3)		52 (58.4)		157 (59.0)		51 (55.4)		155 (53.8)		301 (52.3)		65 (68.4)	
Missing	393	1		0		0		0		0		1		0	
Plurality			0.37		>0.99		0.75		>0.99		0.12		0.094		>0.99
Singleton	91,704 (97.3)	1,281 (97.7)		87 (97.8)		258 (97.0)		90 (97.8)		276 (95.8)		568 (98.4)		93 (97.9)	
Twin or more	2,536 (2.7)	30 (2.3)		2 (2.2)		8 (3.0)		2 (2.2)		12 (4.2)		9 (1.6)		2 (2.1)	
Missing	30	0		0		0		0		0		0		0	
Kotelchuck Index			0.012		0.61		0.013		0.027		0.11		0.11		0.065
Inadequate	9,668 (10.5)	164 (13.3)		10 (13.3)		32 (13.1)		16 (20.0)		41 (14.8)		73 (13.2)		6 (6.9)	
Intermediate	23,561 (25.7)	306 (24.8)		19 (25.3)		62 (25.3)		23 (28.7)		67 (24.2)		124 (22.3)		31 (35.6)	
Adequate	44,236 (48.2)	564 (45.8)		38 (50.7)		98 (40.0)		32 (40.0)		123 (44.4)		272 (49.0)		33 (37.9)	
More than Adequate	14,271 (15.6)	198 (16.1)		8 (10.7)		53 (21.6)		9 (11.2)		46 (16.6)		86 (15.5)		17 (19.5)	
Missing	2,534	79		14		21		12		11		22		8	

Table B displays the number of births with a defect in a single organ system versus multiple. Births with central nervous system most often have defects in one or more additional birth defect groups (28.1%), followed by chromosomal (23.2%). Births with gastrointestinal defects were mostly isolated to that group, although 10 births (10.9%) had additional defects within other groups.

Table B. Distribution of births within each group by number of organ system groups with at least one defect present.

Group, n (%)	Total	Single	Multiple
Any defect[a]	1,311 (100.0)	1,230 (93.8)	81 (6.2)
Central nervous	89 (100.0)	64 (71.9)	25 (28.1)
Circulatory/ respiratory	266 (100.0)	223 (83.8)	43 (16.2)
Gastro-intestinal	92 (100.0)	82 (89.1)	10 (10.9)
Urogenital	288 (100.0)	259 (89.9)	29 (10.1)
Musculo-skeletal	577 (100.0)	529 (91.7)	48 (8.3)
Chromosomal	95 (100.0)	73 (76.8)	22 (23.2)

[a]Birth defect groups will not add up to match the any defect group as some births were reported to have multiple defects

Table C. stratifies the crude and adjusted gastrointestinal model by maternal age. The PR for gastrointestinal defects is no longer significant in mothers <= 25 years of age. However, the PR is increased in mothers > 25 compared to the unstratified models. Among mothers aged > 25, medium exposure to MTR is still insignificant, but the prevalence of gastrointestinal defects was 157% higher in birth records with low active MTR exposure compared to no exposure within 5-km of residence (PR = 2.57; 95% CI = 1.06, 6.25) and 225% higher in births with high exposure compared to no exposure (PR = 3.25; 95% CI = 1.43, 7.36). The wider confidence intervals are most likely due to the reduction in sample size after stratifying the population by age.

Table C. Crude and adjusted relationship between active mountain top removal mining exposure within 5-km of maternal address and presence of a birth defect stratified by maternal age

	Age <= 25		Age > 25	
Model	PR	95% CI	PR	95% CI
MTR exposure crude				
No MTR within 5km	1.00	Reference	1.00	Reference
Low exposure	1.54	0.69 - 3.11	2.58	0.99 - 6.05
Medium exposure	1.20	0.48 - 2.57	2.22	0.80 - 5.40
High exposure	1.36	0.58 - 2.83	3.34	1.41 - 7.40
MTR exposure adjusted[a]				
No MTR within 5km	1.00	Reference	1.00	Reference
Low exposure	1.54	0.73 - 3.25	2.57	1.06 - 6.25
Medium exposure	1.18	0.52 - 2.69	2.22	0.87 - 5.67
High exposure	1.40	0.64 - 3.05	3.25	1.43 - 7.36

PR = Prevalence Ratio; CI = Confidence Interval

[a]Adjusted for maternal education, age, tobacco use, diabetes comorbidity, Kotelchuck index, plurality, and sex of the infant
